# Preservation of Fertility Potential for Gender and Sex Diverse Individuals

**DOI:** 10.1089/trgh.2015.0010

**Published:** 2016-01-01

**Authors:** Emilie K. Johnson, Courtney Finlayson

**Affiliations:** ^1^ Division of Urology, Ann & Robert H. Lurie Children's Hospital of Chicago, Chicago, Illinois.; ^2^ Department of Urology, Northwestern University Feinberg School of Medicine, Chicago, Illinois.; ^3^ Center for Healthcare Studies, Northwestern University Feinberg School of Medicine, Chicago, Illinois.; ^4^ Division of Endocrinology, Ann & Robert H. Lurie Children's Hospital of Chicago, Chicago, Illinois.; ^5^ Department of Pediatrics, Northwestern University Feinberg School of Medicine, Chicago, Illinois.

**Keywords:** disorders of sex development, fertility preservation, gender dysphoria, transgender

## Abstract

Gender and sex diverse individuals—transgender individuals and those with disorders of sex development (DSD)—both face medical treatments that may impair biological fertility potential. Young DSD patients also often have abnormal gonadal development. Fertility preservation for these populations has historically been poorly understood and rarely addressed. Future fertility should be discussed with gender and sex diverse individuals, particularly given recent advances in fertility preservation technologies and evolving views of fertility potential. Key ethical issues include parental proxy decision-making and uncertainty regarding prepubertal fertility preservation technologies. Many opportunities exist for advancing fertility-related care and research for transgender and DSD patients.

## Introduction

The term “gender and sex diversity” refers to (1) transgender individuals for whom gender identity is incongruent with birth-assigned sex and (2) individuals with differences (disorders) of sex development (DSD), also known as intersex, for whom development of chromosomal, gonadal, or anatomic sex is atypical.^1^ In the Lurie Children's Gender and Sex Development Program, we have found that care for one group can inform the other and patients benefit from specialists who are familiar with both. Although many distinctions exist between transgender individuals and individuals with DSD, an important area of commonality lies in the area of fertility—the ability to have a biological child.

Many medical and surgical therapies available to transgender adolescents/young adults (AYA), and children and AYA with DSD, can affect future fertility. Individuals with DSD frequently also have subfertility related to their medical condition. Gender and sex diverse individuals often choose to build a family through options such as adoption or surrogacy. For some, however, it may also be important to have the option of a biological child. Fertility preservation technologies (methods of storing eggs, sperm, or precursor reproductive tissue for future use) and societal attitudes toward “nontraditional” family structures have expanded significantly in recent years such that having a biological child is now possible for many transgender adults and adults with DSD.

The purpose of this perspectives article is to provide an introduction and overview of preservation of biological fertility potential for gender and sex diverse individuals. We also propose next steps for advancing this new clinical and research focus.

## Fertility Preservation for Transgender Patients

Loss of biological fertility was once thought to be a given consequence of gender transition.^2^ Transgender individuals and their healthcare teams have now begun to recognize that options for preserving reproductive potential exist. A majority of transgender adults believe that fertility preservation should be discussed with and offered to them.^3,4^ The Endocrine Society Guidelines recommend counseling for transgender individuals regarding fertility before initiating gender-affirming hormonal therapy.^5^ Knowledge of the effects of medical treatments on fertility, familiarity with available fertility preservation options, and an appreciation for potential ethical issues are all necessary to effectively counsel patients.

### Medical treatments—effects on fertility

Pubertal suppression with gonadotropin releasing agonists (GnRH-a) not only prevents development of potentially distressing secondary sex characteristics but also suspends germ cell maturation. Puberty appears to progress normally after discontinuation.^6^ However, many transgender individuals initiate gender-affirming hormone therapy concurrently with pubertal suppression,^7^ and thus, germ cells never fully mature. This highlights the need for discussion of fertility options before initiating GnRH-a treatment.

Gender-affirming hormones produce impairments in gonadal histology that can cause infertility. Estrogen use by transgender women results in impaired spermatogenesis and an absence of Leydig cells in the testis.^8,9^ Testosterone use by transgender men causes ovarian stromal hyperplasia^10,11^ and follicular atresia.^10^ Gonadal effects of gender-affirming hormones are thought to be at least partially reversible. For example, pregnancy has been reported in transgender men who have previously used testosterone.^12,13^ Thresholds have not been established for the amount and duration of exogenous testosterone or estrogen exposure necessary to have a permanent negative effect on fertility.

For patients who elect surgical transition, gonadectomy will render them permanently sterile. Thus, this is the last opportunity for fertility preservation for patients who choose gender reassignment surgery that includes gonadal removal.

### Fertility preservation options

Table 1 summarizes current fertility preservation techniques, stratified by pubertal status. Transgender individuals who have undergone spontaneous biological puberty consistent with their birth-assigned sex may elect for fertility preservation before initiation of gender-affirming hormone therapy. For transgender women, this involves sperm preservation, with a sample typically obtained through masturbation. When this is not possible due to anatomic, emotional, or physical maturity concerns, sperm can be retrieved directly from the testis using a surgical procedure, testicular sperm extraction (TESE). For transgender men who have undergone female puberty, fertility preservation options include hormonal stimulation followed by oocyte or embryo cryopreservation.

**Table 1.** **Current Fertility Preservation Options**

Gamete type	Pre- or peripubertal options	Postpubertal options
Oocyte	Ovarian tissue cryopreservation^a^	Ovarian stimulation, with oocyte retrieval and cryopreservation of oocyte or embryo
Spermatozoa	Testicular tissue cryopreservation^a^	Sperm banking, with sample obtained through masturbation or surgical biopsy

^a^ Experimental.

Ovarian tissue cryopreservation (OTC), an experimental procedure in which prepubertal ovarian tissue is harvested and preserved, is offered to women with cancer.^14^ OTC is a potential area of future investigation for peripubertal affirmed males, as it does not require hormonal stimulation. Similarly, testicular cryopreservation may offer fertility potential for peripubertal affirmed females. Prepubertal tissue cryopreservation protocols for oncology patients could be expanded to transgender individuals in the future. Although the technology is still developing, fertility experts expect that precursor egg and sperm cells will soon be able to be matured and used with assisted reproductive technologies to produce a biological child.^15–17^

### Ethics

A primary ethical concern regarding fertility preservation for transgender AYA is that parents must make decisions about fertility preservation if it is to occur before initiation of pubertal suppression and/or gender-affirming hormone therapy. Although this is done with patient assent, children are often not intellectually or emotionally mature enough to predict their future desires or understand the implications of their present decisions. This results in transgenerational fertility decision-making, which may not align with future patient desires; disagreement between the patient and parent regarding fertility preservation may also arise. In addition, there may be patient distress associated with procurement processes, given that the gamete type is not congruent with gender identity. Furthermore, peripubertal tissue cryopreservation is experimental. It is unknown if and when assisted reproductive technologies will be available to mature germ cells. Thus, patients are potentially incurring surgical risk and storing gonadal tissue without the guarantee of future fertility.

## Fertility Preservation for Patients with DSD

As for transgender patients, infertility or sterility has often been assumed for many DSD patients. The approach to DSD care has shifted in the last few decades from the assumption that individuals were psychosexually neutral and early sex reassignment surgery was preferred, without concern for future fertility, to one in which it is acknowledged that the formation of gender identity is poorly understood and that irreversible surgeries should be avoided or performed only with great caution and counseling. In current practice, the future fertility potential is considered and discussed, but there remain little evidence and infrequent counseling about fertility preservation.

### DSD conditions—fertility effects

The term DSD refers to a diverse group of conditions that result in atypical sex development. This includes sex chromosome abnormalities, including Turner and Klinefelter syndrome, disorders of gonadal development, including complete and partial gonadal dysgenesis, and disorders of androgen synthesis or action—complete and partial androgen insensitivity (CAIS, PAIS), congenital adrenal hyperplasia, and 5-alpha reductase deficiency. These diverse DSD conditions differ in etiology and fertility potential. DSD patients are at risk for potential infertility or sterility due to the following: (1) abnormal gonadal development, (2) gonadectomy for malignancy risk, (3) abnormal hormonal function, and (4) discordance between gonadal type and gender identity. These often overlapping etiologies are outlined in Table 2, along with description of further required advances in research to better predict fertility potential and applicable preservation techniques.

**Table 2.** **DSD Fertility Effects, Research Needs, and Fertility Preservation Techniques**

Characteristic	Fertility effect	Research needs	Applicable fertility preservation technique
Abnormal gonads (streak, dysgenetic)	Congenital and/or progressive gonadal failure	Establish presence and quality of germ cells by age	Cryopreserve testicular or ovarian tissue^a^
Risk of gonadal malignancy	Gonadectomy traditionally performed	-Malignancy risk-stratification by condition -Surveillance protocols if gonads retained	If gonadectomy required -Postpubertal: preserve sperm or oocytes -Prepubertal: cryopreserve nonmalignant tissue
Abnormal hormone production	Abnormal sperm and oocyte production	Determine fertility rates	Existing assisted reproductive techniques: ovarian stimulation, testicular sperm extraction
Discordance between gender identity and germ cell type	Reframing view allows for biological fertility potential	Patient/parent perspectives needed	N/A

^a^ Experimental.

DSD, disorders of sex development.

### Fertility preservation options

Currently, the treatment for infertility in DSD is limited. General options for pre and postpubertal fertility preservation are the same as for transgender patients (Table 1). However, the inherent gonadal abnormalities faced by DSD patients make the possibility of fertility preservation less certain. Most reports of success in achieving biological offspring are in males, resulting from TESE and/or intracytoplasmic sperm injection; this has been reported in Klinefelter syndrome, PAIS, and 5-alpha reductase deficiency.^18–21^

### Ethics

Ethical concerns related to proxy decision-making, patient maturity, and the experimental nature of prepubertal cryopreservation mirror those that exist for families of transgender individuals. Unique to DSD, some families are now opting to postpone gonadectomy for fertility potential purposes, with an unclear trade-off in terms of gonadal malignancy risk. Researchers are still working to define the fertility potential of many DSD conditions, so counseling regarding the balance between surgical risk, risk of malignancy, and possibility of future fertility is occurring in context of great clinical uncertainty. Finally, many DSDs are a result of genetic mutations, which could be passed to the offspring. Thus, genetic counseling is crucial, and preimplantation genetic diagnosis will play an important role in fertility preservation for DSD patients.

## Conclusions

Transgender and DSD patients are largely disparate, but the medical expertise necessary to care for both overlaps, and the potential for infertility is shared. Both groups have previously been told infertility or sterility was an eventuality. Advancing scientific techniques and broadening perspectives can change this assumption. Table 3 compares key fertility issues facing transgender and DSD patients.

**Table 3.** **Comparison of Fertility-Related Issues for Individuals with Transgender Versus DSD Conditions**

	Transgender	DSD
Inherent gonadal abnormality	No	Frequent
Medical treatments leading to infertility	Frequent	Infrequent
Surgical treatments leading to infertility	Generally only in adulthood	Frequent in childhood
Parental proxy decision-making	Yes	Yes
Medically “unnecessary” surgery for pre- or peripubertal fertility preservation	Yes	Rare
Concern about transmission of a genetic condition	No more than the general population	Yes
Gonadal type discordant with gender identity	Always	Sometimes

Fertility preservation for individuals with gender and sex diversity is a new frontier that requires research and collaboration. Priorities include delineating the fertility desires of patients and families, gaining a comprehensive understanding of treatment effects on fertility potential, further defining the gonadal potential of the diversity of DSD conditions, and expanding possibilities for reproductive tissue preservation and maturation. We must establish research protocols, develop clinical tools to guide fertility-related care, and broaden the medical community's knowledge of current and future fertility preservation possibilities.

## Abbreviations Used

AYA: adolescents/young adults
CAIS: complete androgen insensitivity
DSD: disorders of sex development
GnRH-a: gonadotropin releasing agonists
OTC: ovarian tissue cryopreservation
PAIS: partial androgen insensitivity
TESE: testicular sperm extraction
